# Cell type-specific interactions induce tonic interferon production in endothelial cells in a pathogen-independent manner

**DOI:** 10.1186/s12964-026-02650-4

**Published:** 2026-01-13

**Authors:** Timothy Surette, Fiamma Serra, Ulfert Rand, Tobias May, Luka Cicin-Sain, Mario Köster, Dagmar Wirth

**Affiliations:** 1 Model Systems for Infection and Immunity, Helmholtz Centre for Infection Research, Braunschweig, Germany; 2 Department of Human and Animal Cell Lines, Leibniz-Institute German Collection of Microorganisms and Cell Cultures, Braunschweig, Germany; 3 Department of Viral Immunology, Helmholtz Center for Infection Research, Braunschweig, Germany; 4 Inscreenex GmbH, Braunschweig, Germany; 5 Centre for Individualized Infection Medicine, a joint venture of the Hannover Medical School and Helmholtz Center for Infection Research, Hannover, Germany; 6 German Centre for Infection Research (DZIF) Hannover-Braunschweig site, Braunschweig, Germany; 7 Department of Experimental Hematology, Hannover Medical School, Hannover, Germany

**Keywords:** Endothelial cells, Tonic interferon, SARS-CoV-2, Gap junction

## Abstract

**Background:**

The endothelium promotes a non-adherent vascular surface that facilitates tissue perfusion, prevents clotting, and limits inflammation. Endothelial cells (ECs) execute these tissue-specific functions through the integration of signaling pathways promoted by growth factors, cytokines, extracellular matrix components, and signals from mechanosensory complexes. Furthermore, ECs secrete various molecular signals, leading to the establishment of a specific niche microenvironment. Importantly, ECs can serve as sentinels against invading viral pathogens, propagating anti-viral responses such as the secretion of type I interferons (IFNs). Identification of mechanisms that alter immunity and inflammation at this critical barrier is important to understanding endothelial dysfunctions and the endothelium’s overall role in disease.

**Methods:**

To investigate the regulation and function of IFN signaling in endothelial cells, we used a conditionally immortalized human cell line. We analyzed IFN gene expression by RT-qPCR and used an Mx2 promoter-dependent bioassay to quantify the levels of secreted IFN during homeostatic conditions. Multiple cell types were screened for the ability to enhance tonic IFN production by endothelial cells in a direct coculture model. The role of direct cell-cell interactions in this behavior was studied using cell culture insert settings and inhibitors specifically targeting gap junction communication. The antiviral effects of endothelial tonic IFN production were determined with SARS-CoV-2 and HCMV infections.

**Results:**

We demonstrate that endothelial cells can generate a type I IFN response in absence of infection under homeostatic conditions. These tonic IFN levels rise dramatically when endothelial cells are in direct contact with epithelial cells, though not when cultured with other cell types. The transcriptional induction of type I IFN genes occurs only in endothelial cells and requires direct cell-cell contacts. We further show that IFN induction can be blocked by interfering with gap junction communication and is partially dependent on the cGAS/STING pathway. Notably, the IFN activity derived by this cell type-specific interaction efficiently protects neighboring lung epithelial cells against SARS-CoV-2 infection.

**Conclusions:**

We propose that the upregulation of tonic IFN production by the endothelial-epithelial cell axis can contribute directly to pathogen defense and/or strengthens the innate immune response by elevated priming. While the contributing molecular signaling pathways underlying this activation have not been fully identified, this newly described mechanism has potential relevance during acute or chronic lung injuries, as it enhances the level of tonic antiviral activity. Furthermore, excessive lung inflammation in nonviral pathologies may be dampened by elevated levels of tonic IFNs.

**Supplementary Information:**

The online version contains supplementary material available at 10.1186/s12964-026-02650-4.

## Background

The vasculature plays a crucial role in transporting nutrients, immune cells, and signaling molecules throughout the body. At the same time, it is exploited by pathogens for their dissemination, often acting as a barrier between infectious agents and vulnerable tissues. For this reason, the endothelium has an array of mechanisms to respond to pathogens, staging both an intracellular defense as well as promoting resistance in neighboring cells [1].

To detect and counteract pathogens, endothelial cells are equipped with a wide range of pattern recognition receptors (PRRs), including cytosolic and membrane bound toll like receptors (TLRs), NOD-like receptors (NLRs), retinoic acid-inducible gene-I (RIG-I), and Cyclic guanosine monophosphate-adenosine monophosphate synthase/Stimulator of interferon genes (cGAS/STING) [2]. These PRRs detect and respond to pathogen associated molecular patterns (PAMPs) or damage associated molecular patterns (DAMPs). The endosomal TLRs TLR3, TLR7/8, and TLR9 defend against intracellular pathogens, being activated by double stranded RNA, single stranded RNA, and unmethylated double stranded DNA, respectively [3]. The cGAS/STING pathway is triggered when the cGAS protein encounters cytosolic dsDNA, resulting in the production of cGAMP (or cyclic GMP-AMP) and subsequent activation of the STING protein [4]. Overall, the induction of these PRR pathways initially leads to a plethora of pro-inflammatory and anti-pathogenic effects. This innate immune reaction is characterized by its extremely quick and pathogen-independent response, which is crucial to bridge the time until the highly specific and potent adaptive immune system kicks in [5].

One of the key pathways induced by innate immunity is the type I interferon (IFN) signaling pathways. Type I and type III IFNs are essential components of the innate immune response against viral and other pathogens, mediating immediate defense mechanisms and supporting adaptive immunity. The majority of all cells express the canonical receptor for the type I IFN pathway; IFN alpha and beta receptor (IFNAR). This receptor is a type I transmembrane protein, consisting of two main subunits, a large alpha chain (IFNAR1) and smaller beta chain (IFNAR2). Binding of the receptor’s ligand causes associated JAK kinases (TYK2 and JAK1) to cross-phosphorylate each other and both receptor subunits. These ligands are most commonly IFN alpha or beta (IFN α/β). The activated IFNAR complex then recruits and phosphorylates STAT proteins (canonically STAT1/2/3), which then homo- or heterodimerize and translocate to the nucleus. Once there, the STAT dimers promote the transcription of IFN-stimulated response elements (ISRE). This induces the expression of IFN stimulated genes (ISGs), fostering usually a pro-inflammatory, anti-pathogenic environment both within the cell and for its neighbors. Some cells stimulated by type I IFN will translate and secrete IFN molecules of their own. These proceed to bind autologous IFNAR receptors and the receptors of neighboring cells, magnifying the signal [6]. The type III IFN pathway, consisting of the receptor IFNLR1 and its ligand IFN-λ, serves a similar function to type I IFN, but triggers a less intense response [7].

However, several mechanisms of pathogen-independent IFN activation have been proposed to be triggered by processes such as cell stress, damage, or metabolic changes. These may play roles in launching immune defenses in preparation of pathogen encounter, but may also contribute to pathological processes associated with IFN, such as fatigue, myalgia and depressive disorders. Thus, it is crucial to better understand how IFNs are activated in absence of infection.

It was demonstrated that, under homeostatic conditions in vivo, low levels of IFN are detectable. This was designated as tonic, homeostatic, or constitutive IFN. These low levels were shown to stimulate the IFN response cascade to a certain extent. While usually not being sufficient for defense, this constant, low-level activation can have a “priming” effect on immune cells. This allows the cells to prepare for full activation of the IFN induced response, thereby improving their reactivity to pathogenic challenges [8]. The mechanism has been mainly studied in relation to the microbiome, with commensal bacteria appearing to have an immunomodulatory effect via IFN pathways, thereby contributing to the efficient counteraction against viruses [9, 10]. Tonic IFN shows a distinct cell-type dependent expression pattern and was demonstrated in splenic stromal cells from the red pulp, but not other stromal subsets in the same organ, contributing to early responses to mouse cytomegalovirus (MCMV) infection [11]. In addition, it has been demonstrated that tonic IFN is released even in absence of infection-associated triggers. A study by Ciccarese et al. highlighted the role of IFN in suppressing angiogenic functions of endothelial cells, which was reversed by small interfering RNA knockdown of STAT1 signaling, suggesting tonic IFN production [12]. Tonic IFN signaling was shown to be regulated by the same intracellular pathways that trigger canonical IFN signaling, with the cGAS/STING and TLR pathways having been investigated in this regard [9].

In this study, we investigated the role of tonic IFN expression in endothelial cells. By blocking type I signaling we demonstrate that homeostatic cultures of endothelial cells are characterized by the activation of interferon-stimulated genes (ISGs) and a certain level of protection from infection. Moreover, in contact with epithelial cells, endothelial cells produce elevated levels of IFNs, which was partially dependent on activation of the cGAS/STING pathway as well as the presence of gap junction communication. Finally, we demonstrate that by this mechanism endothelial cells can protect epithelial cells from SARS-CoV-2 infection.

## Materials and methods

### Cell lines and culture conditions

HuARLT cells are a conditionally immortalized human endothelial cell line with preserved functions and were derived from human umbilical vein cells (HUVECs) (designated HuARLT-1 in [13]). These cells require doxycycline (2 µg/mL) for proliferation. HUVECs were obtained from LONZA (Cat# C2519A, Lot# 13TL163012) and used between passages 3 and 5. Endothelial cells were grown in supplemented PromoCell endothelium media (C-22010) with SupplementMix (C-39215) in flasks pre-coated 25% gelatin solution (2% BioReagent tissue culture grade gelatin, type B, in PBS) for > 1 h. Immediately prior to seeding, coating solution was aspirated and surface was washed with PBS.

Immortalized human bronchial basal epithelial cells (BroBECs) were obtained from InScreenEx (CI-huBroBEC, INS-CI-1025) and were grown in huBroBEC medium (INS-ME-1033) with supplement (INS-ME-1033BS). For BroBEC monocultures and cocultures, wells were coated with collagen coating solution (INS-SU-1017-100) for > 1 h. In experiments involving coculture between endothelial cells and BroBECs, collagen coating was used for all conditions.

HeLaMx2Luc cells were derived from HeLa cells by transfection of a bacterial artificial chromosome (BAC RP24-71I6) encoding luciferase under the control of the murine interferon-stimulated Mx2 promoter [14]. They were cultured in Gibco DMEM + 4.5 g/L D-Glucose, L-Glutamine (Ref# 41965-039), 10% FCS, 1% HEPES (1 M), 1% SIGMA Sodium Pyruvate Solution (S8636). When not in an experiment, they were kept under selection by supplementing media with 400ng/mL G418. HeLa-Mx2Luc cells were cultured in gelatin coated flasks.

Immortalized pulmonary artery smooth muscle cells (PASMC) were established by Inscreenex according to a previously described protocol [15]. The cells were grown in 1:1 mix of Thermo Fisher Ham’s F12 Nutrient Mix (Cat# 11765054) and Dulbecco’s modified eagle medium (Ref# 41965-039), supplemented with 5% FCS.

THP-1 leukemic monocyte line was grown in Gibco RPMI Medium (Ref# 21875-034), 10% FCS, and 1% glutamine. To differentiate THP-1 cells, RPMI media was supplemented with 200nM PMA for 48 h prior to the experiment. Primary macrophages were derived from induced pluripotent stem cells and were kindly provided by Nico Lachmann, MHH. iPSC-derived macrophages were grown in Gibco RPMI (Ref# 21875-034) supplemented with macrophage colony-stimulating factor 1 (MCSF, P09603).

CI-HuARLO cells are an immortalized human alveolar epithelial cell line established by Inscreenex (INS-CI-1031) [16]. They were grown in HuAEC medium (INS-ME-1013) in plates coated with collagen coating solution (INS-SU-1017-100) for > 1 h.

A549 are adenocarcinoma human alveolar basal epithelial cells. Cells were cultured in Gibco DMEM + 4.5 g/L D-Glucose, L-Glutamine, 10% FCS, 1% HEPES (1 M), and 1% SIGMA Sodium Pyruvate Solution.

All cells were grown at 37 °C and 5% CO_2_. Unless otherwise specified, all culture plates and flasks were from Thermo Scientific and all media was supplemented with 6.06 mg/mL ampicillin and 10 mg/mL streptomycin. In coculture conditions, cells were grown in a 1:1 mix of their respective media.

### Coculture with cell culture inserts

All cocultures with cell culture insert were performed in Corning deep 24-well plate (Ref# 3527) with Costar 6.5 mm inserts with polycarbonate membrane and pores of 5 μm diameter (Ref# 3421). For contact-free cocultures with cell culture inserts, HeLaMx2Luc cells were seeded at 2 × 10^5 cells/well in a 24-well plate. In separate plates, HuARLT were seeded at 1 × 10^5 cells/well in cell culture inserts and allowed 12 h to attach. Cell culture inserts were then transferred over HeLaMx2Luc monocultures in fresh media. For direct coculture, HuARLT were seeded on inverted cell culture inserts in a 100µL drop of media. This was given 1 h to attach at 37 °C and 5% CO_2_. Cell culture inserts were then placed right-side up in 24-well plate in fresh media and HeLaMx2Luc were seeded on the remaining side of the cell culture insert. Coculture was maintained for 24 h. HeLaMx2Luc cells were then lysed with 1x lysis reporter buffer and analyzed via luciferase assay protocol.

### Cell treatments

Cells were treated with the indicated concentrations of human IFN-β (PeproTech #300-02BC). BroBECs were stained with 1µM calcein violet AM (abcam) or calcein-AM (Cayman) for 1 h. Ruxolitinib was used at a final concentration of 1µM (Biomol, AG-CR1-3624). STING inhibitor C171 (MOLNOVA, M24023) was added at a concentration of 1µM for 24 h. TLR7/8/9-I (MOLNOVA, M28232) was added to cells at a concentration of 1.4µM for 24 h. Oleamide was dissolved from a crystalline solid form in 96% ethanol at a concentration of 100mM and diluted into 37 °C media to the working concentration of 100µM. Media containing oleamide was kept consistently warm to prevent precipitation of oleamide.

### Viruses and infections

The SARS-CoV-2 isolate hCoV-19/Croatia/ZG-297-20/2020 was used, originally described in [17]. All experiments with infectious SARS-CoV-2 were performed in the BSL-3 facility at the Helmholtz Centre for Infection Research (Braunschweig, Germany). SARS-CoV-2 was expanded using Caco-2 cells and stored after harvesting at -80 °C. SARS-CoV-2 titers were quantified by plaque assay using VeroE6 cells as previously described [18]. BroBEC monocultures and HuARLT-BroBEC cocultures were infected with SARS-CoV-2 at an MOI of 29. Cells were fixed with 4% paraformaldehyde in PBS for 20 min at room temperature, followed by permeabilization with 0.1% Triton X-100 in PBS for 10 min and blocking with 1% bovine serum albumin (BSA) in PBS. Antibody staining was performed with mouse anti-SARS-CoV-2 nucleocapsid antibody (SinoBiological) and secondary antibody anti-mouse Cy3 (Dianova). Cocultures were stained with Alexa Fluor 488-labeled Mouse Anti-Human CD31 (BD Pharmingen). Images were captured using the ZEISS 980 Airyscan 2 microscope with a 10x/0.45 Plan-Apochromat objective. Quantitative analyses were performed using ImageJ software (NIH, United States) with appropriate threshold settings. For each imaged region, NP positive area and epithelial area (total area minus CD31-positive area) was manually measured. NP^+^ area was calculated with the formula (NP^+^ Area / Total Epithelial Area) x 100. It is noteworthy to indicate that this protocol underestimates the actual number of infected cells: first, it relies of restrictive threshold values to calculate the area of NP-positive cells. Second, it does not consider the reduced NP positive area of cells that round up or lyse and detach, particularly at later time points.

Human cytomegalovirus mNeonGreen (HCMV-mNG), strain TB40/E, is described elsewhere [19]. Near-confluent cells were infected with HCMV-mNG in unsupplemented EGM. Plates were centrifuged at 800 RCF for 10 min at the beginning of the incubation period. Inoculum was then removed and replaced with fresh media. Efficiency of infection was determined via flow cytometry (BD LSR II flow cytometer) by mNeonGreen detection. In cocultures, endothelial cells were identified using Biolegend BrilliantViolet 421 mouse anti-human CD31 antibody (Ref# 303123).

Generation of the VSV*ΔG(Luc) replicon is described elsewhere [20, 21]. For transduction, endothelial cells were grown in 48-well plates until they reached 80% confluency. Transduction efficiency was quantified 20 h posttransduction by measuring the activity of firefly luciferase in cell lysates (in Relative light units, RLU) using a Berthold Technologies Lumat LB 9507 luminometer. Mock controls were generated by treating cells with fresh mixed media. RLU were normalized to the protein content (RLU/mg Protein) using Peqlab Spectrophotometer ND-1000 Nanodrop machine and Nanodrop 1000 V3.8.1 software.

### Generation of IFNAR-KO endothelial cell line

IFNAR-KO was generated from HuARLT cells using L40C-CRISPR.EFS.mNEON-hIFNAR1-sgRNA1 with GCGGCTGCGGACAACACCCA, based on the vector L40C-CRISPR.EFS.mNEON described in [22]. HuARLT cells were permeabilized via electroporation, using the GenePulser Xcell (BioRad). A cell suspension with a concentration of 1 × 10^6^ cells/mL was prepared in OptiMEM. Cells were transfected in 0.4 cm cuvettes with either 8–16 µg of sgRNA plasmid. Immediately after plasmid addition, each cuvette received 1 pulse of 250 V for 20ms using the square wave protocol. Cells were then reseeded in T25 flasks and cultured normally. 72 h later, a single cell sorting was performed using the FACSAria flow cytometer (BD). Two 96-well plates were seeded with sorted GFP-positive single-cells for creating clonal lines, with the remaining GFP-positive cells being seeded together as a bulk-population.

### RNA extraction and analysis

For isolation of RNA, cells were trypsinized, washed 2x in PBS, resuspended in buffer RLT with β-mercaptoethanol (143mM). RNA was extracted using the QIAGEN RNeasy Mini RNA extraction kit protocol with DNase digestion. RNA quality check, library preparation, and bulk RNA sequencing analysis were performed by the Helmholtz Genome Analytics (GMAK) facility. Quality of RNA was determined using Agilent Technologies 2100 Bioanalyzer. Library preparation used a ribosomal RNA depletion method, targeting both protein-coding and long noncoding RNA. The Thermo Fisher Dynabeads mRNA DIRECT Micro Purification Kit was used, with 500ng total RNA per sample. Libraries were sequenced with an Illumina NovaSeq 6000 machine, using the NovaSeq 6000 S1 Reagent Kit (100 cycles, paired end run) with an average of 5 × 10^7^ reads per sample. Sequencing data resulted in a FASTQ file. Before alignment. FASTQ files were trimmed on base call quality and sequencing adapter contamination using fastq-mcf tool. Reads shorter than 15 bp were removed. Trimmed reads were then aligned to the reference human genome using the open source short read aligner STAR. RNA counts were normalized using trimmed mean of M-values (TMM) method.

**Fig. 1:**
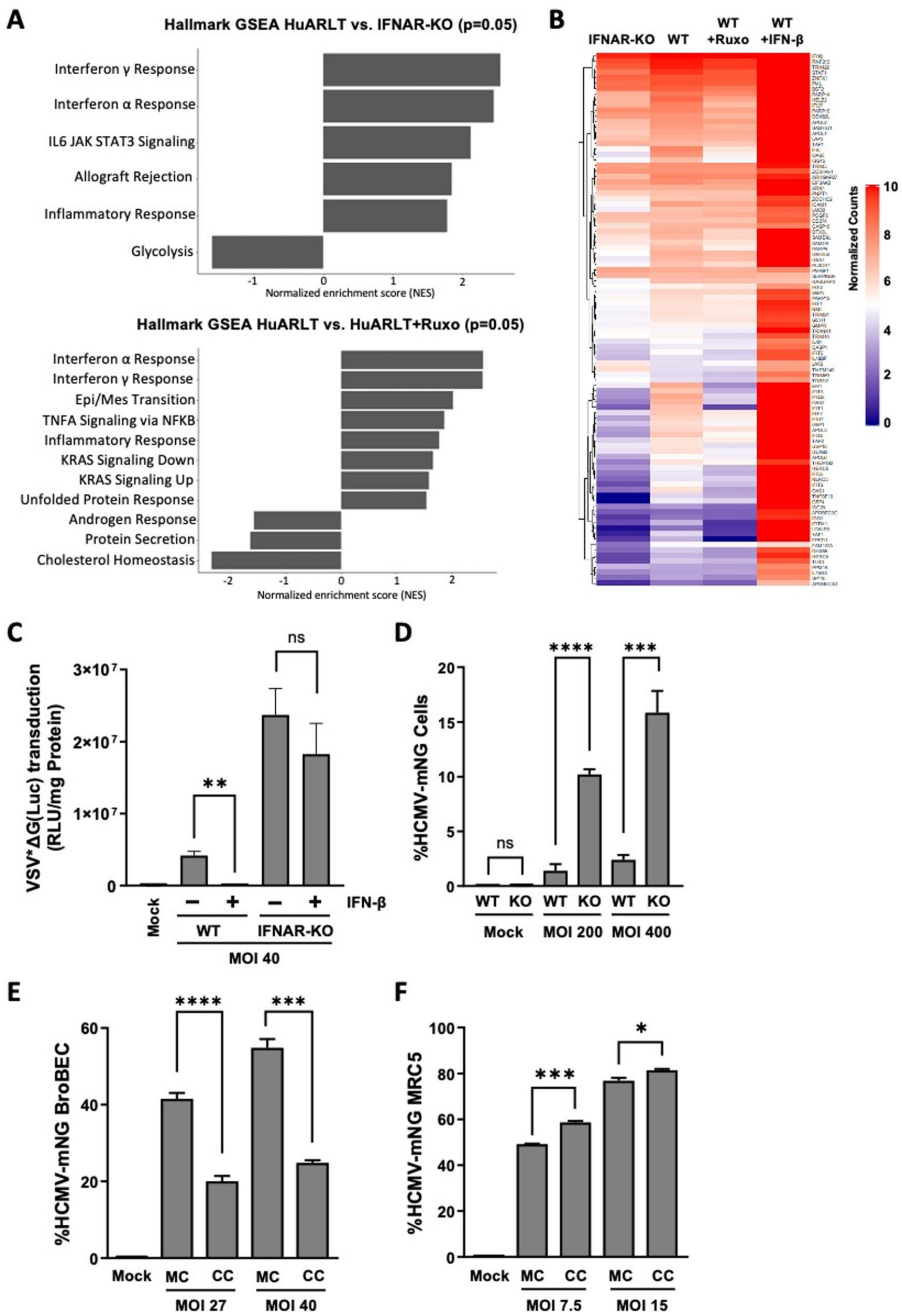
Consequences of blocking type I IFN response. **A** Transcriptome analysis of IFNAR-KO and wild type HuARLT cells with and without 24 h ruxolitinib treatment. Figures show significantly upregulated (*p*</=0.05) pathways in HuARLT cells compared to the ruxolitinib treated and IFNAR-KO cells following gene set enrichment analysis (GSEA). Input for GSEA used difference of normalized RNA counts. **B** The expression of 97 key human ISGs from transcriptome analysis in A is presented in a heatmap. The respective ISG levels of HuARLT cells upon treatment with 500pg/ml IFN is included as control. **C** IFNAR-KO and HuARLT (WT) cells were infected with replication deficient, luciferase encoding VSV (VSV*ΔG(Luc)) at an MOI of 40. Luciferase activity of infected cells indicates successful viral entry. Interferon treated control received 200pg/mL IFN-β for 24 h prior to infection. **D** IFNAR-KO (KO) and HuARLT (WT) cells were infected with HCMV-mNG at MOI of 200 and 400. 72 hpi, the percentage of GFP positive cells was determined via flow cytometry. BroBECs (**E**) and MRC-5 cells (**F**) were grown in monoculture (MC) or in coculture (CC) with HuARLT cells for 24 h. After this period, cells were challenged with HCMV-mNG at indicated MOIs. 72 hpi, the percentage of GFP positive cells was determined via flow cytometry. Note that HuARLT cells were not infected at the low titers used in **E**, **F**. * = *p*-value < 0.05, *** = *p*-value < 0.001, **** = *p*-value < 0.0001, ns = *p*-value > 0.05

The list of human interferon stimulated genes was sourced from Shaw et al. [23]. From this publication, data was taken from supplemental excel file “S1 data”. From this file, only human genes from the worksheet tab “Fig. 1A” were considered. This list was further trimmed by excluding genes with Log2FC values less than 2.5, resulting in a list of 266 genes. This list was then trimmed based on expression in the bulk RNA sequencing data, resulting in a final list of 97 ISGs. Key cGAS STING genes were sourced from the “CGAS-STING signaling pathway” on WikiPathways (https://pfocr.wikipathways.org/figures/PMC10037406__12276_2023_965_Fig1_HTML.html). From a list of 34 genes, a final panel of 14 genes were present in the bulk RNA sequencing data. Heatmaps were generated using the Bioconductor plugin ComplexHeatmap on the R programming language.

Quantitative real-time PCR (RT-qPCR) was performed using the primer pairs for interferon-alpha2 (hIFNA2-fwd: 5’-GCTGAATGACCTGGAAGCCT-3’; hIFNA2-rev: 5’-CTGCTCTGACAACCTCCCAG), interferon-beta (hIFNB1-fwd: 5’-TGCAGCAGTTCCAGAAGGAG-3’; hIFNB1-rev: 5’-AGTCTCATTCCAGCCAGTGC-3’), interferon-lambda1 (hIFNL1-fwd: 5’-TAGCGAGCTTCAAGAAGGCC-3’; hIFNL1-rev: 5’-TGGTCTAGGACGTCCTCCAG-3’), and human beta-actin (hACTβ-fwd: 5’-TCTTCCCCTCCATCGTG-3’; hACTβ-rev: 5’-TTCAGGGTGAGGATGCC-3’). Solutions were prepared using BioRad EvaGreen Supermix. Roche LightCycler 480 machine was used, the analysis was performed using LightCycler 480 SW program (v1.5.1). Normalized crossing points were generated using AbsQuant 2nd Derivative Max method.

### Flow cytometry and FACS

For flow cytometry, endothelial cells were stained with Alexa Fluor 488 Mouse Anti-Human CD31 (BD Pharmingen), while GFP-tagged HeLa cells were directly visualized. Sorting was performed with a BD FACSAria and data was analyzed with FlowJo software (v10.9.0).

### Immunofluorescence staining

For STAT1-pTyr analysis cells were fixed in a -20 °C solution of 1:1 methanol/acetone for 5 min at room temperature. Methanol/acetone solution was then removed, and wells were allowed to dry. Cells were washed 2x with PBS and blocked > 14 h at 4 °C in PBS with 3% bovine serum albumin (BSA). After 2x washing with PBS containing 0.1% saponin, cells were incubated > 14 h at 4 °C with rabbit-IgG anti-STAT1-pTyr (Cell Signaling Technology, #9167), diluted 1:200 in 0.1% saponin solution. After repeated washings, cells were incubated for 45 min with goat-IgG anti-rabbit-Cy3 (Dianova), anti-hCD31-mlgG-AlexaFluor 488 (BD Pharmingen), and Hoechst 33,258 diluted 1:800, 1:250, and 1:500 respectively in 0.1% saponin solution. Cells were then washed 3x in PBS/0.1% saponin solution. For detection of activated STING protein, cells were fixed with 4% paraformaldehyde, followed by permeabilization with 0.1% Triton X-100 and blocking with 1% BSA. Antibody staining was performed with rabbit Phospho-STING (Ser366, E9A9K) antibody (Cell Signaling Technology, #50907, 1:1400 dilution and 16 h incubation at 4 °C) and secondary antibody anti-rabbit Cy3 (Dianova). Images were captured using the ZEISS 980 Airyscan 2 microscope with a 63x/1.40 Oil Corr M27 objective.

### Quantification of IFN using HeLaMx2Luc reporter cells

Diluted cell supernatants were transferred to HeLaMx2Luc cells for 24 h. Cells were washed with PBS, lysed in lysis reporter buffer (Promega). IFN production in cocultures of HeLaMx2Luc and other cell types was assessed upon lysis of cell cocultures in 1x reporter lysis reporter buffer. Luciferase activity in cell lysates (in Relative light units, RLU) was determined using Berthold Technologies Lumat LB 9507 luminometer. Baseline controls were generated by treating HeLaMx2Luc monocultures with fresh mixed media in the cocultures. RLU were normalized to the protein content (RLU/mg) using Peqlab Spectrophotometer ND-1000 Nanodrop machine and Nanodrop 1000 V3.8.1 software.

### Statistics

Significance calculations were performed using GraphPad Prism 9.4.1 (La Jolla, CA, United States). Comparisons between two groups were performed using a parametric, unpaired t-test with Welch’s correction. Comparisons between multiple groups were performed using an ordinary one-way ANOVA, using the Dunnett test. Data with a p value of ≤ 0.05 were considered statistically significant.

## Results

### Endothelial cells exhibit homeostatic type I IFN signaling which conveys protection against HCMV

To characterize tonic, pathogen-independent IFN signaling in human endothelial cells, we compared the transcriptome of homeostatic cultures of HuARLT cells, a HUVEC-derived cell line [24], as well as a type I IFN signaling deficient subclone, which was generated via CRISPR-Cas9-mediated knockout of IFNAR1 (IFNAR-KO). Gene set enrichment analysis (GSEA) revealed that the “hallmark interferon alpha response” as well as partially overlapping hallmarks such as “interferon gamma response” and “inflammatory response” were among the most enriched of the significantly downregulated pathways in the IFNAR-KO cells compared to the HuARLT cells (Fig. 1A). In line with this, treatment of HuARLT cells with the potent JAK1/2 inhibitor ruxolitinib revealed similarly deregulated hallmark pathways (Fig. 1A). Next, we specifically analyzed the RNA levels of 97 ISGs in IFNAR-KO cells and ruxolitinib treated HuARLT cells in comparison to non-treated HuARLT cells. Differential gene expression analysis revealed that 52 (54%) and 28 (29%) of ISGs were more than a 1.2 fold decrease in IFNAR-KO cells or ruxolitinib treated HuARLT cells, respectively (Fig. 1B). The differentially expressed genes include a number of key ISGs such as IFIT1/2, IFITM1, XAF1, and OAS1/2, with a high overlap among the two conditions (Supplemental Fig. 1A/B). Together, these data demonstrate the expression of ISGs as a consequence IFNAR1-dependent signaling under homeostatic culture conditions in endothelial cells.

We asked if this basal activation of ISGs in HuARLT cells is sufficient to convey an antiviral effect. To this end, we infected the cells with a recombinant, luciferase encoding VSV mutant which is capable of entry, but is amplification defective. This limits the infection to a single cycle. Notably, HuARLT cells showed significantly lower luciferase levels than IFNAR-KO cells, while there was no luciferase activity detected if cells were pretreated with 200pg/ml IFN (Fig. 1C). Pre-treatment with 1µM ruxolitinib reversed this protection in the HuARLT, leading to a near 3 fold increase in infection compared to the non-treated HuARLT (Supplemental Fig. 1C). This demonstrates that homeostatic cultures of HuARLT cells are partially protected from infection in a JAK/STAT dependent manner.

To evaluate the consequences of IFN signaling upon infection with a replication competent virus, we challenged IFNAR-KO as well as wild type HuARLT cells with an endothelial cell-tropic strain of HCMV (TB40/E), labeled with mNeonGreen (mNG). 48 h post infection, we measured the mNeonGreen signal by flow cytometry. The percentage of infected IFNAR-KO cells was near 7 fold higher compared to HuARLTs, at both multiplicity of infection (MOI) values (Fig. 1D). This suggests that the homeostatic ISG levels in HuARLTs are also sufficient to confer a certain level of protection against HCMV infection.

Next, we explored whether this protection could also be conveyed to neighboring cells. Thus, HuARLT cells were cocultured with human lung bronchial basal epithelial cells (BroBECs), followed by infection with HCMV-mNG. Two days after infection, the cocultured cells were analyzed by flow cytometry and the fraction of mNG-positive BroBECs was determined. If compared to monocultures, cocultured BroBECs were significantly protected against HCMV infection, with around a 3 fold reduction at both MOI values (Fig. 1E). Notably, coculture of HuARLT cells with MRC5 fibroblasts provided no significant protection to HCMV infection (Fig. 1F), indicating that endothelial cells can convey protection against viral infection to cocultured cells in a cell-type dependent manner.

### Endothelial-epithelial coculture increases tonic IFN production

**Fig. 2:**
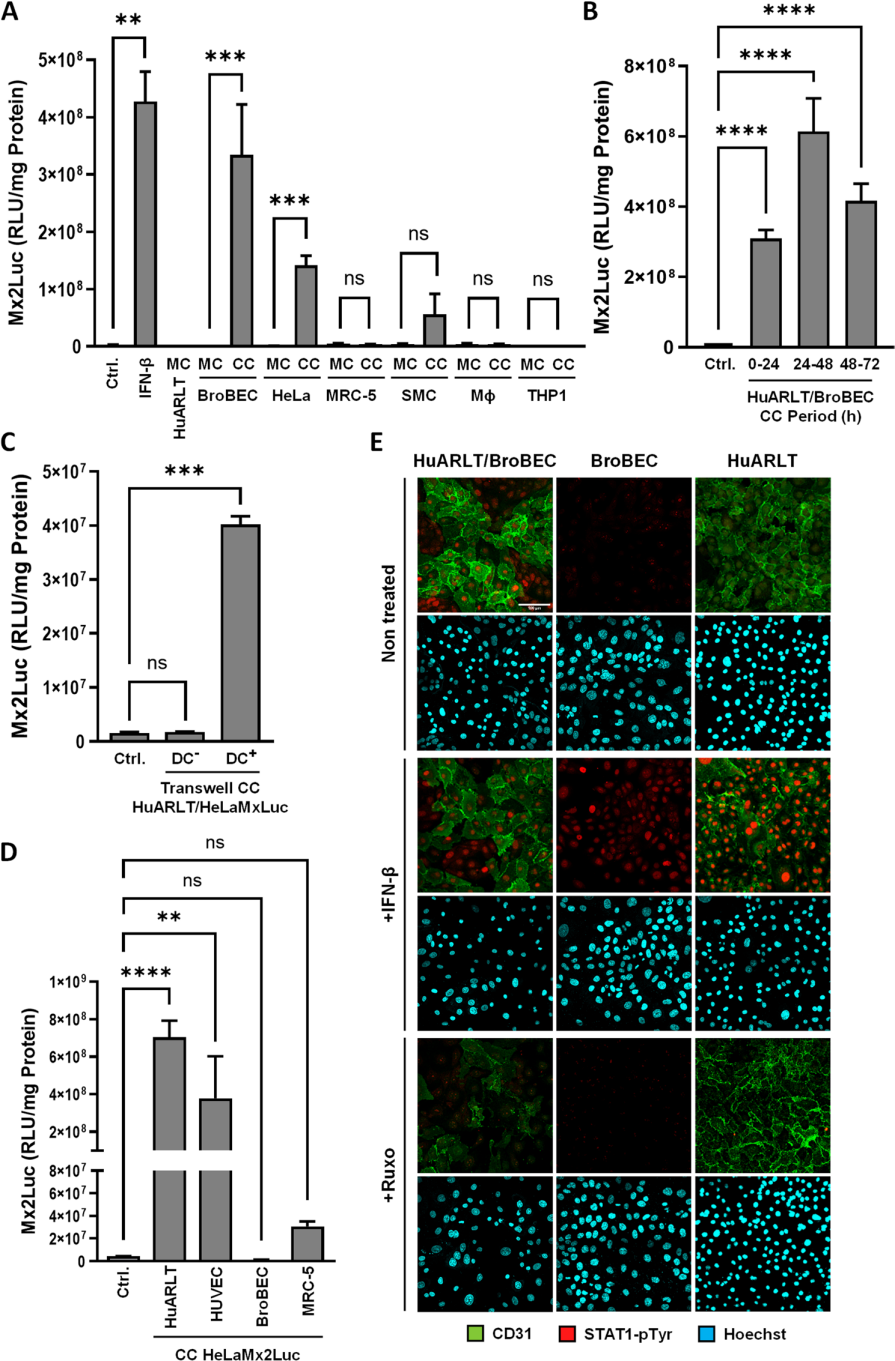
Endothelial/epithelial cocultures show increased IFN activities. **A** HuARLT cells were grown in monoculture (MC) or 1:1 coculture (CC) with BroBECs, HeLa cells, MRC-5 cells, PASMCs (SMC), primary macrophages (MΦ), and THP-1 cells. The IFN activity in supernatants was quantified on HeLa-Mx2Luc reporter cells. IFN-β control samples were generated by treating reporter cells with 20pg/mL IFN-β for 24 h. Control (Ctrl) shows baseline of reporter cells in fresh media. **B** Figure shows luciferase activities in supernatants from a 72 h coculture between HuARLT cells and BroBECs (1:1). Supernatants were collected every 24 h and evaluated on HeLaMx2Luc reporter cells. **C** HuARLT cells were seeded in 6.5 mm transwell inserts with pore size of 5 μm. After 24 h, the transwell inserts were transferred to a well with HeLaMx2Luc reporter cell monolayers seeded in the bottom (DC-). Direct cocultures (DC+) were generated by subsequent seeding of HuARLT and HeLaMx2Luc cells on opposite sides of a transwell insert. After 24 h of coculture, reporter cells were lysed and luciferase expression was measured. Ctrl refers to the baseline luciferase activity of the reporter cells. **D** HeLaMx2Luc cells were grown in monoculture (Ctrl) or coculture (CC) with HuARLT cells, HUVECs, BroBECs, and MRC-5 cells. After 24 h, cells were lysed and luciferase activity was measured to quantify IFN activity. **E** Immunostaining of pY701-STAT1 (red) in monocultures and cocultures of HuARLT cells and BroBECs upon treatment with IFN-β or ruxolitinib (+ Ruxo). Counterstaining for CD31 (green) was used to visualize HuARLT cells, Hoechst 33,258 staining indicate localization of nuclei. ** = *p*-value < 0.01, *** = *p*-value < 0.001, **** = *p*-value < 0.0001, ns = *p*-value > 0.05

The presence of an IFNAR1-dependent baseline ISG signature in endothelial cells suggests the homeostatic expression of type I IFNs. The variability in antiviral state induction may indicate differential expression depending on the nature of the cocultured cell. Thus, we quantified the levels of IFN activity in the cell supernatant of endothelial cell cultures, either in monoculture or in 1:1 coculture with various other cell types. For quantification we took advantage of a HeLa reporter cell line, in which the prototypical ISG Mx2 controls luciferase and visualizes IFN activity with a sensitivity of 1 pg/ml IFN-β (HeLa-MxLuc, Supplemental Fig. 2A). Despite the transcriptional activation of various ISGs, no specific IFN activity could be detected in the supernatant of HuARLT monocultures, indicating that the IFN levels were below detection limit (Fig. 2A). Unexpectedly, we observed that the supernatant of HuARLT/BroBEC cocultures increased Mx2-driven luciferase activity more than 200 fold compared to the monocultures of both BroBECs and HuARLT cells, corresponding to the activity of approximately 20pg/mL IFN-β (Fig. 2A). Similar to HuARLT, a BroBEC coculture with the IFNAR-deficient endothelial cells resulted in significantly increased IFN levels, though to reduced levels if compared to the wild type HuARLT cells (Supplemental Fig. 2B). To rule out the possibility that the increase in tonic IFN activity is a result of cell stress arising during coculture, we determined the frequency of dead cells after 24 h of coculture or in respective monocultures. Notably, live/dead staining indicated that cell death remained below 1% across all culture conditions. (Supplemental Fig. 2C). We further tested whether doxycycline, which is required to induce proliferation in the HuARLT cells, contributes to IFN production. Actually, the presence of doxycycline had no significant impact on IFN levels in mono- and cocultures of HuARLTs and BroBECs (Supplemental Fig. 2D). Cocultures of HuARLT with another epithelial cell line (HeLa) showed comparable levels of IFN activity, while cocultures with smooth muscle cells (PASMCs) revealed only non-significantly increased IFN levels. Importantly, no significant IFN related activity was detected in the supernatants of HuARLT cocultures with MRC-5 fibroblasts, primary macrophages, and THP-1 monocytes (Fig. 2A), suggesting that increased tonic IFN levels are specifically induced when coculturing HuARLT cells with epithelial cells such as BroBEC and HeLa cells.

To determine the kinetics of the IFN related activity, we collected the supernatants from three consecutive time periods after the start of coculture (0–24 h, 24–48 h, and 48–72 h) and measured the IFN released from cocultures with BroBECs. Strongly enhanced tonic IFN activity was detected in all tested time periods (Fig. 2B). Thus, the generation of an IFN related activity in the supernatant is not restricted to just the early stages of coculture, but rather appears to be a sustained process.

To investigate whether this IFN-related activity depends on cell-to-cell contacts between the endothelial and epithelial cells, we performed coculturing experiments using cell culture inserts. Therefore, HuARLT cells were seeded on a cell culture insert, while a layer of HeLa-MxLuc cells was seeded into the main well. This created a physical separation between the two cell layers while allowing exchange of medium and secreted molecules. As control, HuARLT and HeLa-MxLuc cells were sequentially seeded on the upper and the lower surface of the cell culture insert, respectively, allowing direct intercellular contact through the 5 μm diameter pores of the membrane. After 24 h of coculture, the supernatants of these two conditions were evaluated for IFN activity. Notably, the coculture on opposite sides of the porous transwell insert clearly induced reporter expression, while physical separation of the two cell types did not increase baseline activity in the ISG reporter cells (Fig. 2C), indicating that cell-cell contacts are crucial for this effect. Next, we evaluated the extent of cell-to-cell communication between HuARLT cells and epithelial cells by making use the Calcein dye transfer assay. While non-fluorescent calcein-AM enters cells, it becomes a hydrophilic, fluorescent dye upon cleavage by intracellular esterases. The cleaved dye is unable to escapes cells passively, but can be exchanged with neighboring cells via the formation of junctional channels. We determined the functional coupling of BroBECs, and two other epithelial cell lines, HuARLO [16] and A549, with HuARLT cells. Epithelial cells were labeled with calcein and subsequently cocultured for 6 h with unlabeled HuARLT cells. The proportion of HuARLT cells that take up the calcein label was determined by flow cytometry. Notably, about 80% of HuARLT cells took up the dye from BroBECs over this period, while there was nearly no exchange with HuARLO and only minor exchange with A549 cells (with 4% and 30% calcein positive HuARLT, respectively). This demonstrates the different ability of the cell lines to undergo cell-to cell communication (Supplemental Fig. 2E). Importantly, the ability to form cell-cell contacts correlated with the ability to induce IFN, since BroBECs, but not A549 and HuARLO cells could increase IFN production upon coculture with HuARLT (Supplemental Fig. 2F).

To ascertain if epithelial cells are also able to induce IFN activity in cell types other than endothelial cells, we assessed cocultures of epithelial HeLa-Mx2Luc cells and fibroblastoid MRC-5 cells as well as epithelial BroBECs. Cocultures of HeLa-Mx2Luc cells with HuARLT cells as well as with primary endothelial cells (HUVEC) were used as control. As depicted in Fig. 2D, cocultivation of epithelial HeLa-Mx2Luc cells with both non-endothelial cell lines did not induce IFN activity, while the control cocultures with endothelial HuARLT cells and HUVECs induced high luciferase expression (Fig. 2D).

To confirm that the cells are activated via intracellular JAK/STAT signaling, we performed immunostaining of the HuARLT-BroBEC cocultures for the presence of tyrosine-phosphorylated STAT1 (STAT1-pTyr). As controls, we stimulated monocultures of both cell types, as well as the co-culture with IFN-β. Notably, in the coculture condition, activated STAT1-pTyr was clearly detectable in the nuclei of both BroBECs and HuARLT cells even in the absence of exogenous IFN-β. This activation of STAT1 activation dependent on JAK/STAT signaling since it was completely abrogated by pretreatment of the co-culture with ruxolitinib. In contrast, unstimulated monocultures of both cell types did not show accumulation of STAT1-pTyr in the nucleus, while control IFN-β stimulation caused robust signals of nuclear phosphorylated STAT1 (Fig. 2E). This demonstrates that HuARLT-BroBEC co-cultures are characterized by an activated JAK/STAT signaling cascade.

### Endothelial cells release type I and type III IFNs upon co-culture with epithelial cells

**Fig. 3:**
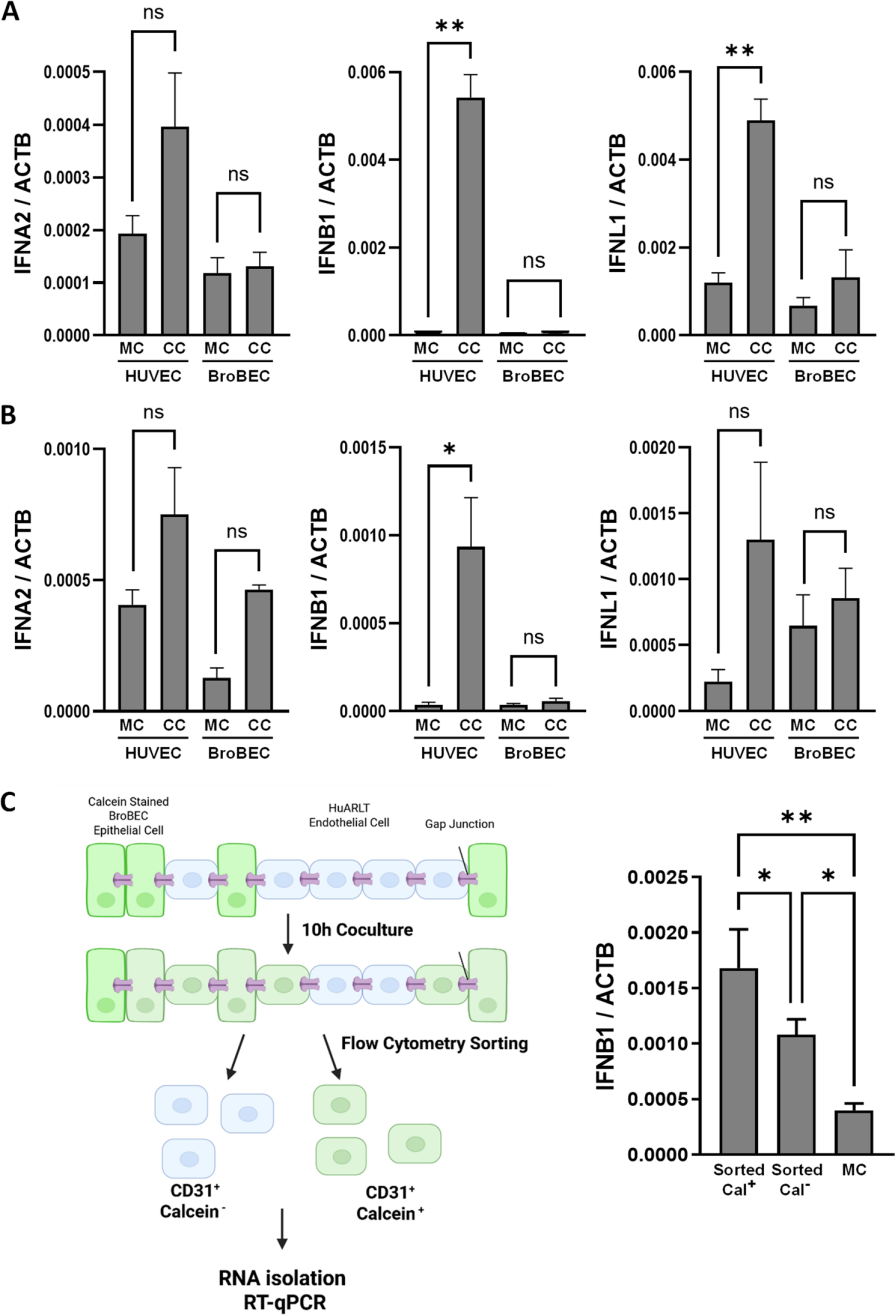
RT-qPCR analysis of IFN genes in mono- and cocultures of endothelial and epithelial cells. **A**, **B** Figures show qPCR analysis of IFNA2, IFNB2 and IFNL1 transcripts in HuARLT cells (**A**) or HUVECs (**B**) and BroBECs grown in either 1:1 coculture (CC) or monoculture (MC) for 20 h. Endothelial and epithelial cells in coculture were separated via FACS upon staining for CD31. Bar graphs show the averaged 2-(ΔCt)values of technical triplicates. **C** The schematic on the left shows the experimental workflow (created in Biorender). The calcein-AM-stained BroBECs and unstained HuARLT cells (1:1) were cocultured for 10 h. Calcein+ and calcein- HuARLT cells were then isolated by FACS, followed by RNA extraction and RT-qPCR analysis. Bar graphs show the averaged 2-(ΔCt)values of biological triplicates. * = *p*-value < 0.05, ** = *p*-value < 0.01, ns = *p*-value > 0.05

To identify the nature and source of IFN related activity in the supernatant of endothelial-epithelial cocultures, we determined mRNA expression of different IFN subtypes by RT-qPCR. To this end, endothelial cells (HuARLT or HUVEC) and epithelial BroBECs were grown in coculture for 18–20 h. Endothelial cells were separated from the epithelial population by CD31 staining and subsequent cell sorting. RNA was isolated from both cell populations and RT-qPCR analysis was performed using specific primers for the IFN subtypes IFN-α2, IFN-β, and IFN-λ1. Respective monocultures were included as controls. Among the three IFN subtypes, IFN-β expression increased strongly in cocultured endothelial HuARLT cells and HUVECs when compared to their monocultures (Fig. 3A and B, respectively). We also observed enhanced expression of IFN-α2 and IFN-λ1 in cocultured HuARLT cells and HUVECs but at a lower level. In contrast, BroBECs did not show an increase of mRNA expression of any of the three IFN subtypes upon coculture with HuARLT cells (Fig. 3A). However, a slight increase in IFN-α2 mRNA levels were obvious in BroBECs upon coculture with HUVECs (Fig. 3B). These results indicate that endothelial cells were the primary source of type I and type III IFNs in coculture with epithelial cells. In agreement with the elevated IFN levels, the IFN-stimulated genes RSAD2 (viperin) and Mx1 were also observed to be increased in HUVECs in coculture with BroBECs (Supplemental Fig. 3A). Further, we observed that under coculture condition expression of the VEGF-dependent, angiogenesis associated genes VCAM, CD34, and VWF was increased in HuARLT cells (Supplemental Fig. 3B), supporting the observation that (low level of) type I IFN signaling does not interfere with endothelial properties but rather primes expression of angiogenesis associated genes [25].

In cocultures, the frequency and extent of contacts between epithelial and endothelial cells can vary. We hypothesized that this should be reflected by differences in IFN-β transcripts in HuARLT cells which establish strong connections with epithelial cells if compared to HuARLTs with minor interactions. To investigate this, HuARLT cells were subjected to coculture with calcein labeled BroBECs. After 10 h of coculture endothelial cells were isolated by CD31 staining. Cell sorting was used to isolate calcein-positive and calcein-negative HuARLT cell populations, reflecting strong and minor levels of cell-cell communications. The IFN-β transcripts in these populations were quantified and compared to HuARLT monoculture. Notably, IFN-β transcripts in the calcein-positive HuARLT population were significantly increased over those from both calcein-negative HuARLT cells and HuARLT monocultures (Fig. 3C). This indicates that the extent of cell-to-cell contacts (reflected by different levels of calcein uptake) correlates with the ability of cells to express IFN-β. Note that calcein-negative HuARLTs showed significantly more IFN-β transcripts than cells in monoculture, suggesting that cell sorting based on the calcein dye did not fully select for endothelial cells isolated from contact with BroBECs. With this, we conclude that endothelial cells that are in contact with epithelial cells show higher levels of IFN-β.

### Cocultures of endothelial cells provide protection against SARS-CoV-2

**Fig. 4:**
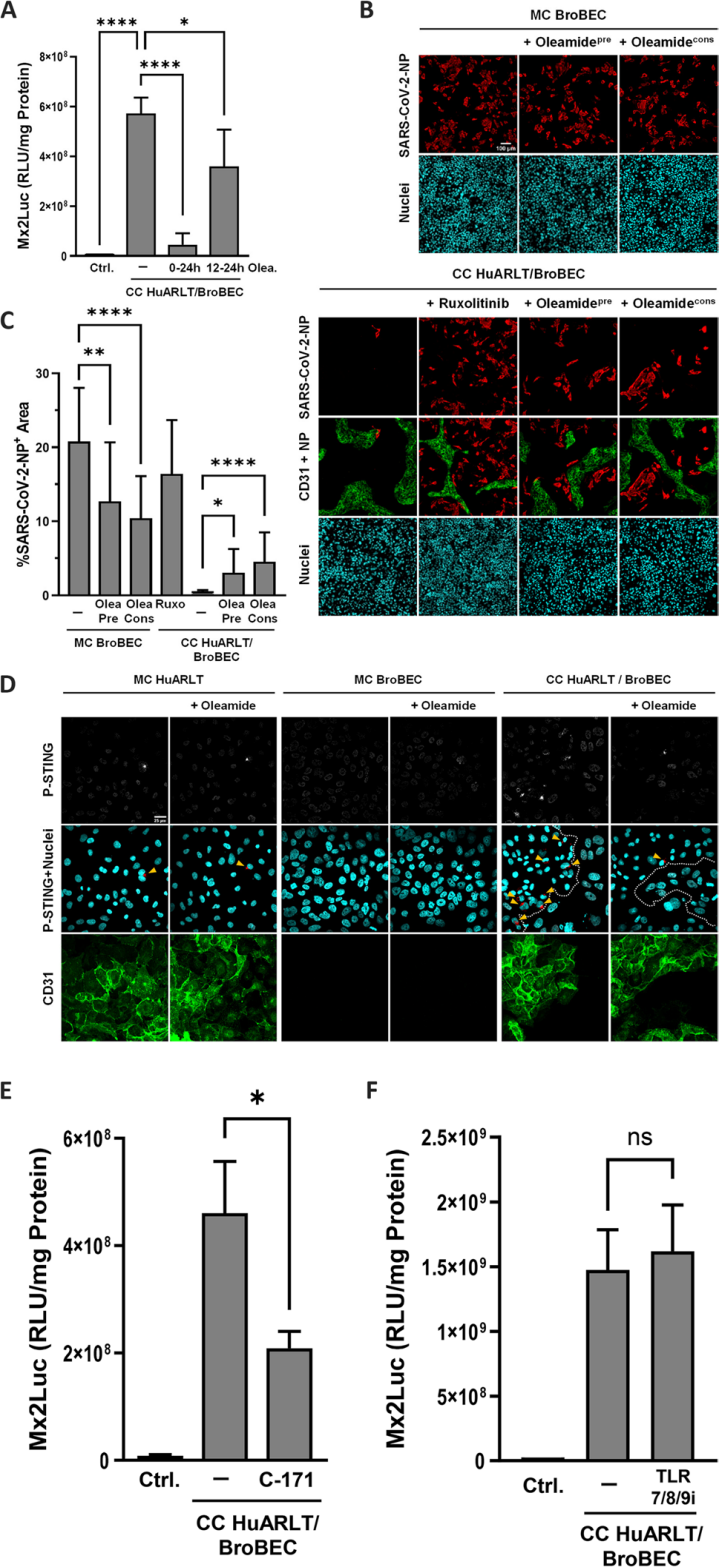
Molecular mechanisms underlying IFN production in cocultures and block of SARS-CoV-2 infection. **A** HuARLT cells and BroBECs were grown in coculture (CC) for 24 h. Oleamide was added either during co-seeding of both cell types (24 h) or 12 h after coculture start (12 h). Control (Ctrl) shows data from reporter cells treated with HuARLT monoculture supernatant. **B** SARS-CoV-2 infection was performed at an MOI of 29 for BroBECs in monoculture (MC) or coculture (CC) with HuARLT cells. Infection is indicated through a SARS-CoV-2-NP stain (red), while HuARLT cells were stained using a CD31 stain (green). Oleamide pretreatment (oleamidepre) cells received 24 h of oleamide treatment prior to infection while oleamide constant (oleamidecons) cells received oleamide before and during the infection. For each image captured, NP+ area was calculated for the quantification of infection in figure **C**. The experiment underlying figures (**B**) and (**C**) was performed three times, with biological duplicates, results of one representative experiment is depicted. At least 18 stochastically selected regions were analyzed for each condition. **D** HuARLT cells and BroBECs were grown in coculture (CC) or in respective monocultures for 24 h. Oleamide treated cells were grown in media supplemented with 100µM oleamide over the (co-)culture period. Immunostaining of activated STING protein was performed with S366-Phospho-STING antibody (P-STING). Counterstaining for CD31 was used to visualize HuARLT cell compartment, Hoechst 33258 staining indicate localization of nuclei. Dotted line in the merged images of P-STING+Nuclei indicate border between endothelial and epithelial cells, while yellow triangles indicate perinuclear accumulation of P-STING. **E**, **F** Figure shows quantified IFN levels in 24 h supernatant from 1:1 HuARLT/BroBEC coculture, using HeLaMx2Luc reporter cells. Treated cells received 24 h of either 1µM C171 STING inhibitor (**E**) or 1.4µM TLR7/8/9-I (**F**), with treatment beginning in parallel with coculture start. The control shows the baseline RLU/measurements of the reporter cell monoculture in coculture media. * = *p*-value < 0.05, ** = *p*-value < 0.01, **** = *p*-value < 0.0001, ns = *p*-value > 0.05

Since the coculture on porous membranes revealed that robust IFN induction depends on the ability to establish cell-cell contacts (Fig. 2C), we asked whether specifically intercellular communication via gap junctions is required to release IFN from endothelial cells. To this end, we investigated calcein transfer in cocultures of dye-labeled BroBECs with HuARLT with or without treatment with the gap junction inhibitor oleamide [26, 27]. Notably, oleamide efficiently reduced the frequency of calcein positive HuARLT cells and BroBECs (Supplemental Fig. 4A). We confirmed that oleamide does not alter IFN-β activation per se, which excludes any unspecific impairment of IFN signaling (Supplemental Fig. 4B). Next, the supernatants of oleamide treated cocultures were assessed for release of type I/III IFNs. HuARLT cells and BroBECs were cocultured in the absence or presence of oleamide for 12 h and 24 h. Supernatants of both conditions conferred a significant reduction of IFN levels as visible by reduced activity when transferred to reporter cells, with the prolonged treatment with oleamide completely abrogating the activation of the reporters (Fig. 4A).

Considering the physiological proximity of endothelial cells and epithelial cells within the lung, we investigated whether elevated tonic IFN production by endothelial cells can limit respiratory virus infection in the bronchial epithelial cells. Thus, we subjected either BroBEC monocultures or cocultures with HuARLT cells to SARS-CoV-2 infection and determined the viral replication in the absence or presence of the gap junction inhibitor oleamide. Viral replication was quantified by staining the viral nucleoprotein (NP) 48 h post-infection. To assess the frequency of infected cells in the different culture conditions, we quantified the NP-positive area within the area of CD31-negative BroBECs. As shown in Fig. 4B/C, monocultures of BroBECs were efficiently infected by SARS-CoV-2, with an average NP-positive area of 20% at 48 h post-infection. Notably, in coculture, SARS-CoV-2 infection of the BroBECs was nearly completely blocked, with only a few NP-positive cells detectable per field-of-view (Fig. 4B/C). To inhibit IFN activity and the subsequent activation of an antiviral state in the BroBECs during coculture, we treated the cells with ruxolitinib before infection with SARS-CoV-2. Importantly, ruxolitinib pre-treatment of the endothelial-epithelial coculture regained infectivity in the BroBEC compartment up to 16% NP-positive area. In agreement with the known specificity of SARS-CoV-2 to epithelial cells, infection under this condition was still largely restricted to CD31 negative epithelial cells. These results show that IFN activity provided *in trans* by the endothelial cells can induce an efficient antiviral state in epithelial cells, controlling SARS-CoV-2 infection.

In agreement with the finding that gap junction inhibition interferes with the release of IFN activity into the supernatant of endothelial-epithelial cocultures, infection of the BroBEC compartment was partially restored in the presence of oleamide. Pre-treatment of cocultures with oleamide for 24 h, as well as the continued administration during the course of infection, resulted in a significant increase in infection frequency to mean values of 3–5% NP-positive area of CD31-negative cells (Fig. 4B**/C**). In contrast, oleamide treatment of BroBEC monocultures resulted in a twofold reduction of the SARS-CoV-2 infection. Together, these findings suggest that gap junctional coupling between endothelial and epithelial cells is a prerequisite to enforce tonic IFN production to levels that protect cells against SARS-CoV-2 infection.

We aimed to shed light on the signaling pathways that are involved in the enhanced tonic IFN production by endothelial cells. Numerous reports have indicated that cytosolic accumulation of self-nucleic acid species, including nuclear DNA fragments as well as mitochondrial DNA, culminates in the activation of the cyclic GMP-AMP synthase (cGAS)-STING pathway [28, 29]. Indeed, we observed accumulation of phosphorylated STING (P-STING) in HuARLT monocultures with a frequency of 2.5% of cells (Fig. 4D, Supplemental Fig. 4C), which is in line with the low level ISG induction (Fig. 1B). In contrast, no activated STING protein was detectable during BroBEC cultivation. Importantly, the frequency of P-STING increased dramatically in HuARLT cells upon coculture with BroBEC cells, while still no activated STING protein was detectable in the epithelial cells (Fig. 4D, Supplemental Fig. 4C). Further, oleamide treatment of HuARLT-BroBEC cocultures reduced the levels of activated STING protein to the levels of endothelial monocultures. Thus, we conclude that the enhanced levels of tonic IFN production during epithelial-endothelial coculture correlate with elevated levels of activated STING and depend on functional gap junction communication. To further investigate if the cGAS/STING pathway is involved in the activation of tonic IFN production, cocultures were treated with the STING inhibitor C171 [30]. Notably, C171 treatment resulted in a significant 50% reduction of IFN activity in the coculture supernatant (Fig. 4E). However, C171 mediated STING inhibition had no significant impact on SARS-CoV-2 infection (Supplemental Fig. 4D), suggesting that this is not the only signaling pathway involved.

TLR9 is the second major DNA sensor to be implicated in the recognition of self-DNA and induction of pathogenic type I IFN responses [31, 32]. Notably, treatment of HuARLT-BroBEC cocultures with the small molecule inhibitor TLR7/8/9-I did not change the levels of IFN activity in the supernatant (Fig. 4F). Together we conclude that changes in cell communication via gap junction channel activity and the STING pathway are two of the main drivers of enhanced tonic IFN production.

## Discussion

The family of type I IFNs fulfills key functions in antiviral and antimicrobial defense in nearly all cell types. Several of its biological functions, including the regulation of innate and adaptive immunity, crucially depend on the rapid and strong upregulation of type I IFN production. However, beyond its antipathogenic defense mechanisms, many reports have highlighted a physiologic role of low and constitutive levels of IFN. This tonic IFN expression has been shown to convey an efficient subsequent response to other cytokines and was shown to be relevant, e.g. for immune and bone homeostasis [33, 34]. Naturally, the ability of endothelial cells to mount an efficient antiviral response is crucial in restricting viral dissemination through the vasculature. However, type I IFN response in the endothelium has a major impact on organ function, e.g. by increasing vascular permeability, inflammatory cell recruitment, and interfering with VEGF-induced angiogenesis [35, 36]. Thus, a detailed understanding of the sources and mechanisms of type I IFN production at endothelial barriers, especially under homeostasis, could help to identify critical factors of endothelial dysfunction.

Our study demonstrates that endothelial cells exhibit a tonic IFN gene signature under homeostatic conditions (Fig. 1A/B). Tonic ISGs are defined as a set of ISGs that are decreased when IFNAR-signaling is abolished in the absence of a pathogenic stimulus [37]. Gene set enrichment analysis revealed that the type I IFN response was significantly downregulated in endothelial cells treated with the JAK/STAT inhibitor ruxolitinib or after CRISPR/Cas9-mediated knockout of type I IFN receptor signaling. Among the top 20 ISGs that showed higher expression in untreated HuARLT cells were several genes associated with antiviral activity such as IFIT1/2, ISG15, OAS1/2 (Supplemental Fig. 1A/B). This evidence of basal IFN signaling in endothelial cells corroborates previous observations by Ciccarese et al. [12] and may account for the reduced susceptibility of HuARLT cells to infection compared with IFNAR-deficient IFNAR-KO cells, as observed both with the replication-incompetent VSV (Fig. 1C) and with HCMV (Fig. 1D). It would be interesting to evaluate if endothelial cells from different tissues similarly show tonic IFN responses.

Strikingly, the ability of endothelial cells to produce tonic IFN drastically increased upon coculture with certain epithelial cells, but not with other cells types that are known to interact with endothelial cells like smooth muscle cells, fibroblast, and macrophages (Fig. 2A). We detected IFN activity corresponding to 20pg/mL IFN-β in the supernatant of endothelial-epithelial cocultures. Notably, in the absence of direct contacts between the endothelial and epithelial cells, this drastic increase in tonic IFN production was not observed (Fig. 2C). Indeed, the absence of intercellular communication, either by inhibiting gap junction formation (Fig. 4A) or in cocultures of cells that do not have the capacity to exchange factors (Supplementary Figs. 2E and 2 F), abrogated elevated IFN levels. Upon coculturing cell-communication-capable BroBEC epithelial cells and endothelial cells, the level of IFN activity was able to strongly reduce infections of epithelial cells with SARS-CoV-2. This conclusion is supported by the restoration of SARS-CoV-2 infections to levels observed in monocultures of lung epithelial cells when endothelial-epithelial cocultures were pretreated with the JAK inhibitor ruxolitinib (Fig. 4C). Further, in homeostatic cocultures of endothelial and epithelial cells, a constitutive accumulation of phosphorylated STAT (pY701-STAT) was detected in the nuclei of both cell types (Fig. 2E). This accumulation was completely reversed by ruxolitinib treatment. Together, these results confirm that type I IFN receptor signaling in the absence of infection plays a major role in establishing an antiviral state under homeostatic conditions. It is interesting to note that IFNAR-deficient endothelial cells produced over 100 fold less IFN upon coculturing with epithelial cells (Supplemental Fig. 1E). This could indicate that, without functional IFNAR signaling, the knockout endothelial cells are not primed by the constitutively released IFN, leading to less robust overall IFN production during the coculture with epithelial cells, compared to wild type cells. The presence of such an autocrine signaling loop mediated by intracellular signaling through the type I IFN receptor may have a role in the interaction of various cells with their microenvironment and control cell type specific susceptibilities towards innate immune responses [38, 39].

We confirmed that endothelial cells were the main source of IFN production during coculture with BroBECs (Fig. 3A/B). Further, we revealed that the amount of endothelial cells positive for activated STING protein increased dramatically upon coculture with BroBECs. Strikingly, blockade of gap junction communication by oleamide resulted in a strong reduction of P-STING positive endothelial cells (Fig. 4D, Supplemental Fig. 4C). In line with this observation, oleamide treatment strongly impaired IFN activity in the coculture supernatant (Fig. 4A) and partially restored SARS-CoV-2 dissemination in the epithelial cell population (Fig. 4B/C). Gap junction proteins, three of which are present in the endothelium (Cx37, Cx40, and Cx43), allow small molecules to pass directly into the cytoplasm of neighboring cells without contacting the extracellular milieu [40]. The second messenger cGAMP, synthesized by the DNA sensor cGAS, was shown to act as a communicator between cells, promoting STING activation and IFN gene induction in cells even when they are not in direct contact with PAMPs [41]. Of note, we observed that STING inhibition reduced the amount of IFN activity released by endothelial cells in coculture conditions (Fig. 4E), suggesting that this pathway contributes to the activation of IFN secretion. However, STING inhibition was unable to rescue SARS-CoV-2 infection in cocultures (Supplemental Fig. 4D), which might indicate that STING activation is not the only driver of antiviral activity directed against SARS-CoV2 infection. Likewise, the absence of enhanced IFN gene expression in the epithelial cells (Fig. 3A/B) and key ISGs (Supplemental Fig. 3A) argues against elevated levels of small molecule STING activators like cGAMP in epithelial cells and their GJ-mediated horizontal transfer to the endothelial cell population. Previously, it was shown that stressed and necrotic epithelial cells release DAMPs or alarmins that alert the immune system and trigger a sterile inflammatory response [42, 43]. In particular, during acute bacterial infections, intestinal epithelial cells can release endogenous danger signals, like the purine nucleotide ATP, through connexin hemichannels which precedes the onset of inflammation [44]. However, the low fraction of dead cells in coculture excludes that cell stress is a relevant factor (Supplemental Fig. 2C).

Thus, it is likely that other factors contribute to tonic IFN induction in endothelial cells. A well-known mediator is calcium. Calcium ions (Ca^2+^) are classical small molecule messengers that can pass freely between gap junctions, has been associated with gap junction induced inflammatory signaling [45]. The regulation of these ions can lead to a mechanism referred to as cell-cell uncoupling, with high Ca^2+^ levels eventually leading to the closure of gap junction gates [46]. Ca^2+^ mediated electrical pulses have been observed to be integral to TLR2-mediated inflammatory signaling in mucosal epithelial cells. When cells were stimulated by inflammatory agents, Ca^2+^ pulses induced inflammatory signaling in neighboring, non-stimulated cells [47]. However, a direct link between IFN signaling and Ca^2+^ gradients has not yet been described.

Another potential mediator could be extracellular ATP. Elevated concentrations of extracellular ATP stimulated angiogenic responses in vasa vasorum endothelial cells [48] which was associated with the co-activation of VEGF receptor-2, expression of Vascular Cell Adhesion Molecule-1 (VCAM-1), and monocyte recruitment in coronary artery endothelial cells [49, 50]. Interestingly, Zhang et al. showed that RAW macrophages and L929 fibroblasts release ATP upon infection with VSV [51]. Furthermore, extracellular ATP was shown to stimulate IFN-β gene expression and subsequent antiviral activities dependent on P2X7 receptor expression, an ATP-gated ion channel. However, while extracellular ATP was found to inhibit STING, TLR-3, and RIG-I mediated interferon signaling in airway epithelial cells [52] the influence of extracellular ATP on endothelial cells remains to be elucidated. Beyond ATP specifically, extracellular nucleic acids have been found to activate IRF3 in a gap-junction dependent manner, although the [53].

A knockout or downregulation of gap junction genes might help to elucidate the specific pathways. However, such experiments are challenging giving the various redundant proteins involved in this type of cell communication. Further experiments are needed to identify what causative agent(s) are exchanged between the endothelial and epithelial cell pools, leading to the observed elevated tonic IFN production.

In various pathologic conditions, the integrity of the basement membrane between the endothelial and epithelial layers is known to become compromised [54]. This may facilitate direct contact of the two cell types. In patients with chronic obstructive pulmonary disease (COPD), respiratory viral infections are more likely to spread to the lower airways, causing exacerbation of the disease [55, 56]. In these situations, damage to the barrier at the lower airways can occur [57]. Moreover, endothelial cell progenitors can contribute to regeneration in injured tissues [58], a process that implies direct cell-cell contacts. Finally, Niethamer et al. showed that following lung damage capillary endothelial cells differentiated into multiple subtypes, some of which were predicted to be contributing to subsequent lung recovery [59]. Indeed, a ligand-receptor interaction analysis indicated that epithelial-endothelial interactions were increased during injury, for all endothelial subtypes [59, 60]. Apart from injury, the epithelial-endothelial axis might play a role in natural processes, e.g. embryonic development. In line with this, Akeson et al. observed endothelial invasion into the alveolar epithelial chambers of mice lungs upon VEGF overexpression [61].

The endothelial cell-derived IFN activity could have diverse biological consequences in vivo. IFN signaling has been previously linked with endothelial dysfunction and angiogenic disruption [12, 62–64], and such endothelial susceptibility or elevated sensitivity may contribute to the pathogenesis of SARS-CoV-2 infection. Indeed, vascular dysregulation has been observed following infection in both acute and long COVID infections [65, 66]. Furthermore, a recent study demonstrated an anti-inflammatory role of type I IFN signaling in the lung [67]. In this study, loss of type I IFN signaling increased inflammatory cell recruitment and elevated inflammatory cytokine responses in nonviral disease contexts were observed. These observations suggest that the anti-inflammatory activity of type I IFN may limit excessive inflammatory responses and thereby mitigate lung injury. Tonic IFN production by the endothelium may extent the pleiotropic effects of IFNs beyond their established antiviral roles.

Elevated tonic IFN production at endothelial-epithelial interfaces may also be relevant in tumors. Enhanced angiogenesis and tissue invasion is a common feature of cancer pathogenesis [68], while IFN signaling has been observed to suppress angiogenesis in tumors [69, 70]. Consequently, tonic IFN activity may serve as an intrinsic regulatory mechanism that counteracts pro-angiogenic and invasive processes within the tumor microenvironment.

While this study provides proof for tonic IFN production from endothelial cells, particularly upon contact with epithelial cells, it has some limitations. Most importantly, the study could not completely unravel the underlying molecular mechanisms. The fact that blocking the cGAS/STING pathway could only partially reduce IFN levels and was not sufficient to block SARS-CoV2 infection hints towards the contribution of other players. Recently, mitochondrial DNA has been identified as an inducer of STING in epithelial cells, giving rise to elevated basal levels of IFN-λ2/3 [71]. However, our observations hint towards a different mechanism in endothelial cells, given the lack of overt STING activation in monocultures of endothelial cells.

Moreover, further studies would be needed to unravel the mechanism by which epithelial cells contribute to the activation of IFN signaling in cocultured endothelial cells. The fact that not all the epithelial cell lines tested show this ability might suggest that this feature can be lost during immortalization or is specific for a certain origin. Evaluation of tonic IFN production from primary cells of different tissues/organs should shed light on the restrictions.

## Conclusion

We propose that homeostatic IFN signaling might represent a previously underappreciated mechanism by which endothelial cells interact with the cells around them and confer protection against infection. This effect appears to be significantly stimulated when interacting with gap-junction competent cells of an epithelial lineage. These findings may further elucidate how the endothelium incorporates type I IFN signaling both in homeostatic tissue regulation and in pathologic situations where the barrier between the epithelium and endothelium is disrupted. Future studies will be required to fully identify the molecular determinants and pathways underlying this pathogen-independent mode of IFN activation across the endothelial-epithelial axis, and to clarify in which physiological or pathogenic scenarios these processes occur in vivo.

## Supplementary Information

Supplementary Material 1.

## Data Availability

No datasets were generated or analysed during the current study.
