# Supplementary material for: Cell type-specific interactions induce tonic interferon production in endothelial cells in a pathogen-independent manner
Source: Cell Commun Signal. 2026 Jan 13;24:54. doi: 10.1186/s12964-026-02650-4 (PMC12849652; doi:10.1186/s12964-026-02650-4)
Supplement: Supplementary file 1 — Supplementary Material 1. [file 12964_2026_2650_MOESM1_ESM.pdf]

Supplemental Figures

Cell type-specific interactions induce tonic interferon production in endothelial cells in a pathogen-independent manner

Timothy Surette, Fiamma Serra, Ulfert Rand, Tobias May, Luka Cicin-Sain, Mario Köster, Dagmar Wirth

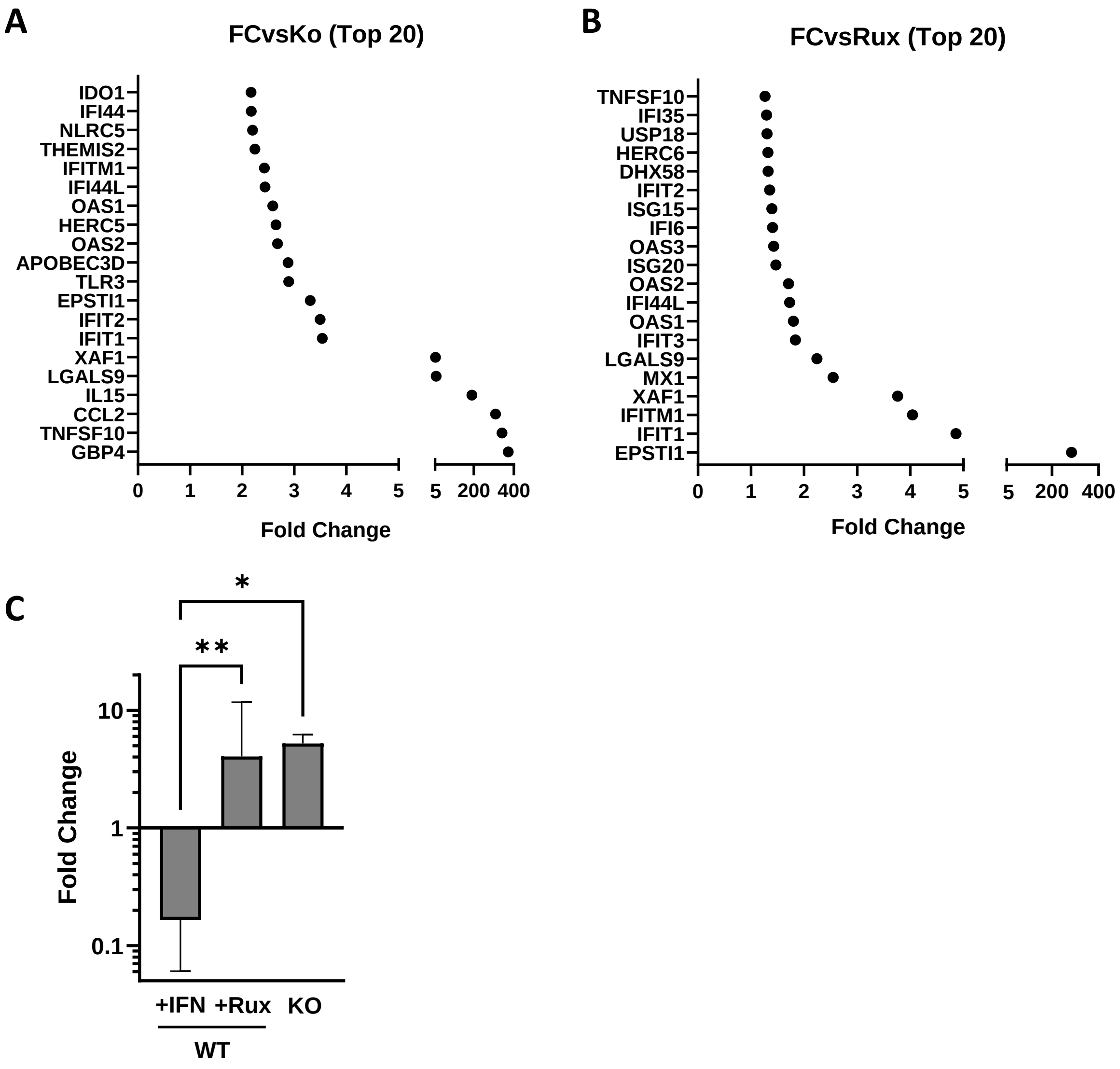

Supplemental Figure 1

A), B) *Top 20 upregulated ISGs in wt-HuARLTs compared to HuARLT-IFNAR-KO or wt-HuARLT treated with ruxolitinib, respectively. Values were based on difference of normalized RNA counts.* ***C)*** *IFNAR-KO (KO) and HuARLT (WT) cells were infected with replication deficient, luciferase encoding VSV at an MOI of 40. Figure shows fold change of averaged RLU/mg protein values relative to luciferase activity of non-treated, wild type HuARLT cells, which is 1. Interferon and ruxolitinib treated control received 200pg/mL IFN-β or 1µM ruxolitinib for 24h prior to infection, respectively.*

(= p-value < 0.05, ** = p-value < 0.01).*

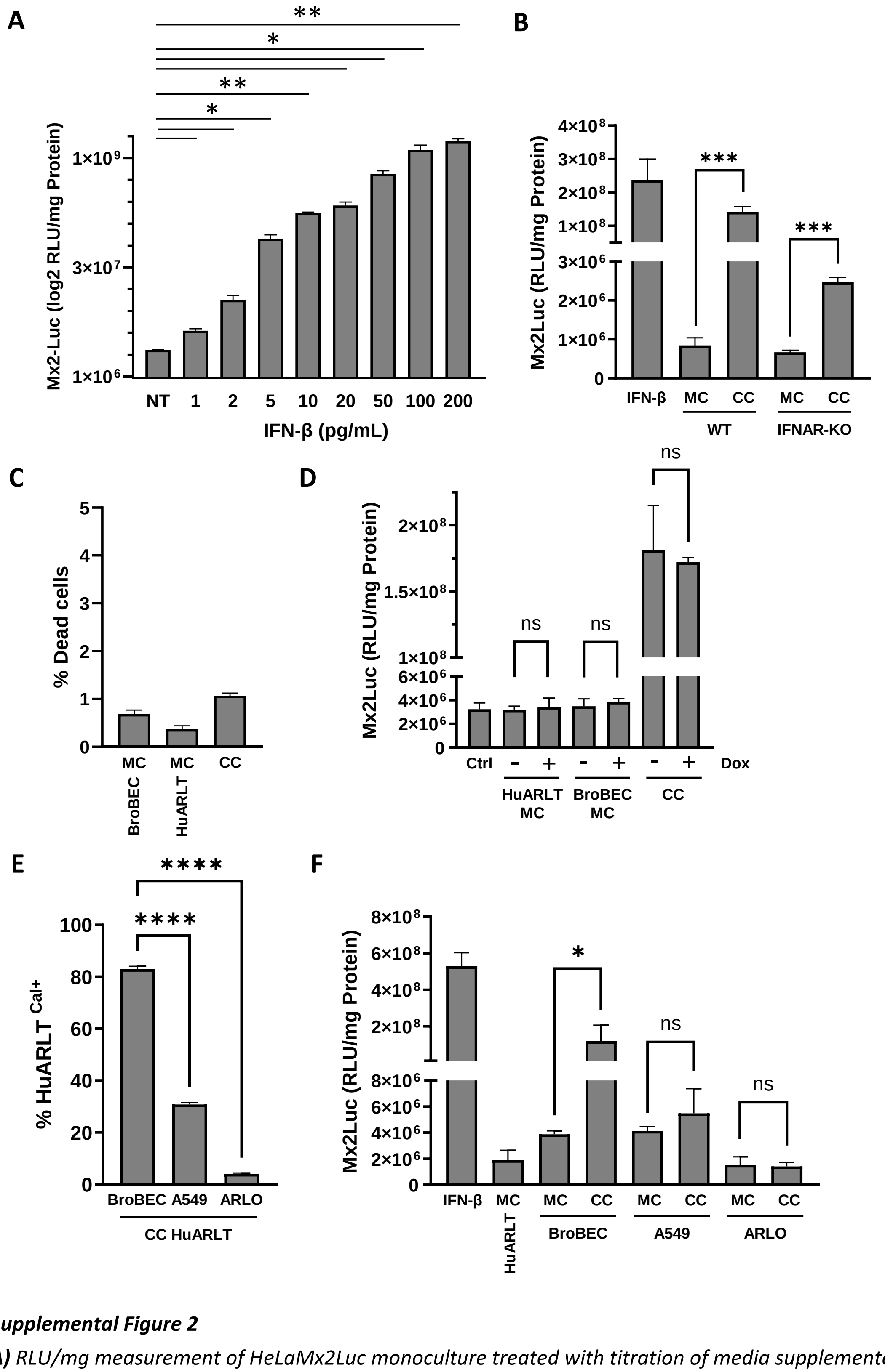

Supplemental Figure 2

***A)** RLU/mg measurement of HeLaMx2Luc monoculture treated with titration of media supplemented with indicated concentrations of h-IFN-β for 24h. **B)** Wt-HuARLT and HuARLT-IFNARko were grown in monoculture or 1:1 coculture with BroBEC. IFN activity in the supernatant was quantified using HeLaMx2Luc reporter cells. IFN control represents*

Mx2Luc activity upon treatment of reporter cells with 20pg/mL IFN-β for 24h. ***C)*** *Near IR live-dead stain of HuALRT cells and BroBECs, either in coculture or monoculture. The percent near IR positive indicates the fraction of dead cells in culture detected via FACS.* ***D)*** *HuALRT cells and BroBECs, either in coculture or respective monocultures, were cultured in the absence or presence of doxycycline supplemented media for 24h. IFN activity in the supernatant was determined by HeLaMx2Luc cells.* ***E)*** *HuARLT cells were grown in coculture for 6h with epithelial cells (BroBEC, A549, or HuARLO) labelled with Calcein. Figure shows the percentage of Calcein-positive HuARLT cells after the coculture period with the different epithelial cell lines.* ***F)*** *IFN activity of supernatants from 1:1 cocultures of HuARLT with BroBEC, A549 or CI-HuARLO. Cells were grown in coculture (CC) or monoculture (MC) for 24h. Supernatants were transferred to HeLaMx2Luc reporter cells and luciferase activity was determined after 24h. IFN represents treatment of reporter cells with 20pg/mL IFN-β.*

(= p-value < 0.05, ** = p-value < 0.01, *** = p-value < 0.001, **** = p-value < 0.0001, ns = p-value > 0.05).*

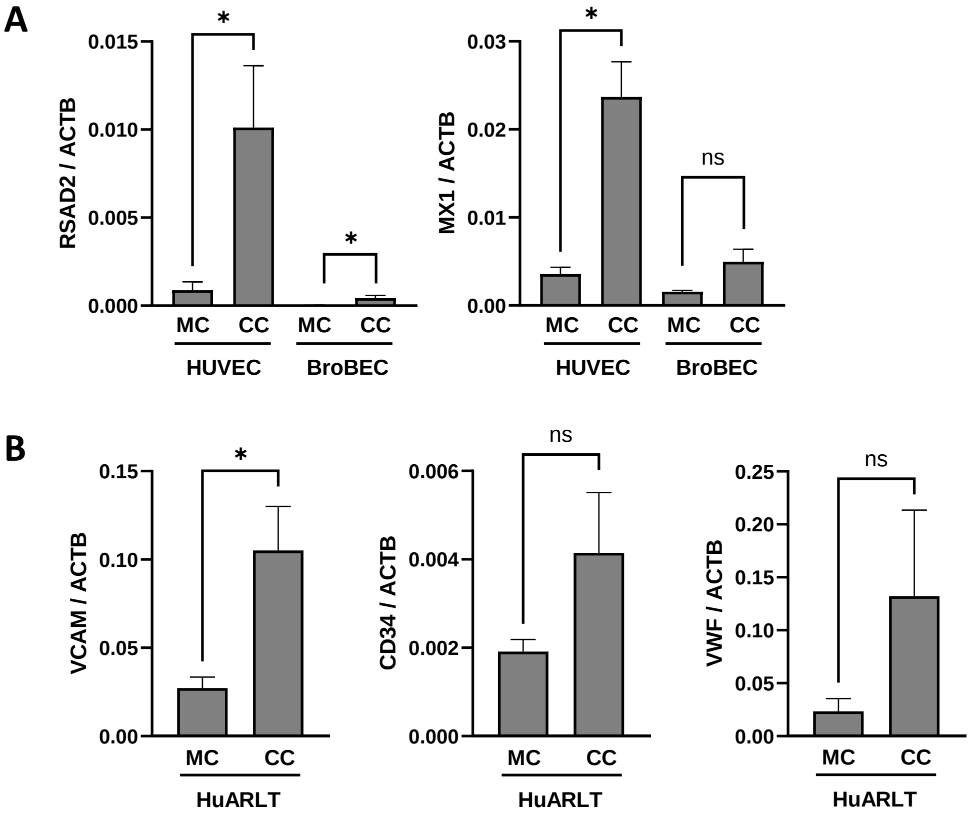

Supplemental Figure 3

A) *Figures show qRT-PCR analysis of ISG transcription, using the genes of RSAD2 (viperin) and MX1. HUVECs and BroBECs were grown in either 1:1 coculture (CC) or monoculture (MC) for 20h. Endothelial and epithelial cells in coculture were separated via FACS upon staining for CD31. Bar graphs show the averaged* $2^{-(\Delta Ct)}$ *values of technical triplicates.* ***B)*** *Figure shows qRT-PCR analysis of endothelial marker transcription, using VCAM, CD34, and VWF in HuARLT cells grown in monoculture (MC) or 1:1 coculture (CC) with BroBECs for 16h. Bar graphs show averaged* $2^{-(\Delta Ct)}$ *values of biological triplicates.*

(= p-value < 0.05, ns = p-value > 0.05).*

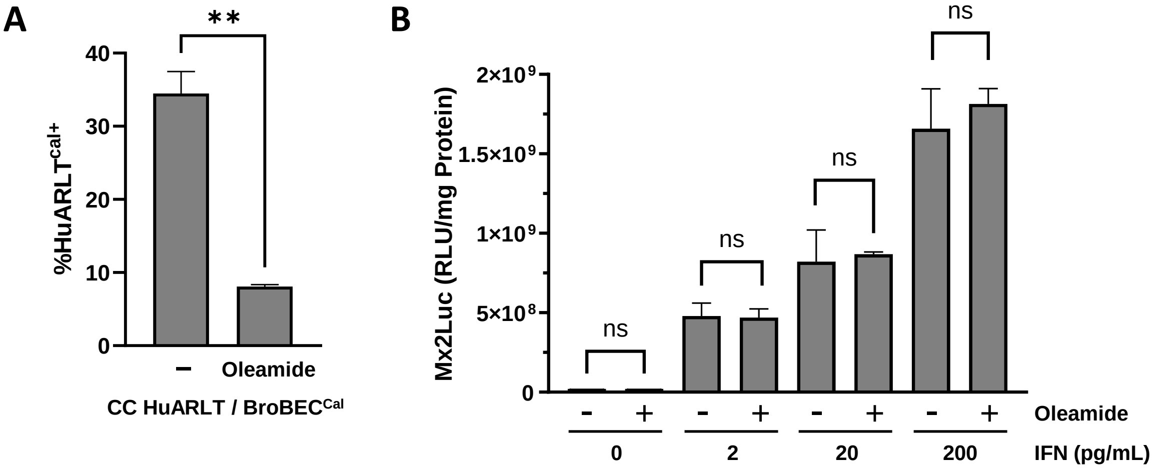

C

	Total number of cells	No. P-STING positive cells	% P-STING positive cells
MC HuARLT control	711	18	**2.5**
MC HuARLT Oleamide	1057	13	**1.2**
MC BroBEC control	866	0	**0**
MC BroBEC Oleamide	689	0	**0**
CC control[1]	499	117	**23.4**
CC Oleamide[1]	635	24	**3.8**

[1] In HuARLT/BroBEC coculture (CC) only CD31-positive HuARLT cells

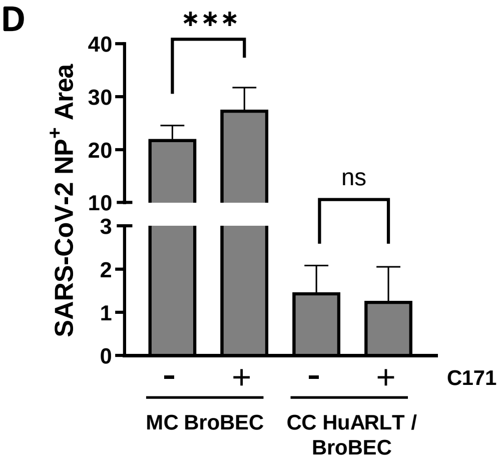

Supplemental Figure 4

A) *HuARLT were grown in coculture for 7h with BroBECs labelled with Calcein, with or without a 4h pre-treatment with 100µM oleamide. Flow cytometry was used to assess the percentage of calcein positive CD31 positive HuARLTs after the coculture in presence or absence of oleamide.* ***B)*** *IFN-β activation in HeLaMx2Luc reporter cells, with and without 24h of 100µM oleamide pre-treatment.* ***C)*** *HuARLT cells and BroBECs were grown in coculture (CC) or in respective monocultures (MC) for 24h. Oleamide treated cells were grown in media supplemented with 100µM oleamide over the coculture period. Antibody staining was used to indicate the presence of activated STING protein (S366 Phospho-STING) and to separate endothelial HuARLT cells from BroBECs (CD31). The number of P-STING positive cells were counted from microscopic images described in* ***Figure 4D****. The table shows percentage of P-STING*

positive cells, normalized against total number of cells for monoculture condition or against the number of CD31-positive endothelial cells in coculture condition. ***D)*** *SARS-CoV-2 infection was performed at an MOI of 29 for BroBECs in monoculture (MC) or coculture (CC) with HuARLT cells. C171 treated cells received the STING inhibitor for 24h prior to infection. Cells were fixed and infection was quantified through a SARS-CoV-2-NP stain. CD31 staining of HuARLT cells was used to normalize the NP-positive area of coculture images to the area of BroBECs. Thus, SARS-CoV2-NP-positive area represents level of infection in the BroBEC compartment.*

*(** = p-value < 0.01, *** = p-value < 0.001, ns = p-value > 0.05).*
